# Overnutrition Induced Cognitive Impairment: Insulin Resistance, Gut-Brain Axis, and Neuroinflammation

**DOI:** 10.3389/fnins.2022.884579

**Published:** 2022-07-06

**Authors:** Qin Zhang, Kangyu Jin, Bing Chen, Ripeng Liu, Shangping Cheng, Yuyan Zhang, Jing Lu

**Affiliations:** ^1^First Clinical Medical College, Zhejiang Chinese Medical University, Hangzhou, China; ^2^Department of Psychiatry, The First Affiliated Hospital, Zhejiang University School of Medicine, Hangzhou, China; ^3^Second Clinical Medical College, Zhejiang Chinese Medical University, Hangzhou, China; ^4^School of Life Sciences, Zhejiang Chinese Medical University, Hangzhou, China; ^5^Key Laboratory of Mental Disorder Management in Zhejiang Province, Hangzhou, China

**Keywords:** obesity, overnutrition, central insulin resistance, gut microbiota, neuroinflammation

## Abstract

Overnutrition-related obesity has become a worldwide epidemic, and its prevalence is expected to steadily rise in the future. It is widely recognized that obesity exerts negative impacts on metabolic disorders such as type 2 diabetes mellitus (T2DM) and cardiovascular diseases. However, relatively fewer reports exist on the impairment of brain structure and function, in the form of memory and executive dysfunction, as well as neurogenerative diseases. Emerging evidence indicates that besides obesity, overnutrition diets independently induce cognitive impairments *via* multiple mechanisms. In this study, we reviewed the clinical and preclinical literature about the detrimental effects of obesity or high-nutrition diets on cognitive performance and cerebral structure. We mainly focused on the role of brain insulin resistance (IR), microbiota-gut-brain axis, and neuroinflammation. We concluded that before the onset of obesity, short-term exposure to high-nutrition diets already blunted central responses to insulin, altered gut microbiome composition, and activated inflammatory mediators. Overnutrition is linked with the changes in protein expression in brain insulin signaling, leading to pathological features in the brain. Microbiome alteration, bacterial endotoxin release, and gut barrier hyperpermeability also occur to trigger mental and neuronal diseases. In addition, obesity or high-nutrition diets cause chronic and low-grade systematic inflammation, which eventually spreads from the peripheral tissue to the central nervous system (CNS). Altogether, a large number of unknown but potential routes interact and contribute to obesity or diet-induced cognitive impairment. The challenge for future research is to identify effective interventions involving dietary shifts and personalized therapy targeting the underlying mechanisms to prevent and improve cognition deficits.

## Introduction

Obesity has increased significantly in prevalence and has become a major global health challenge because of its association with increased heart disease, diabetes, stroke, hypertension, cancer, and mental disorders (Aronne, 2001; Avila et al., 2015; Chao et al., 2019). In recent years, the worldwide proportion of adults with body mass index (BMI) of 25 kg/m^2^ or greater has risen to 36.9% in men and 38% in women (Ng et al., 2014). It is predicted that by 2025, global obesity prevalence will reach 18% in men and surpass 21% in women (2016). Traditionally, obesity is considered one of the major factors of metabolic syndrome (MetS) including insulin sensitivity, hyperinsulinemia, hyperglycemia, and hypertension. Recently, accumulating evidence has shown that obesity leads to neuropsychiatric disorders such as Alzheimer’s disease (AD), Parkinson’s disease (PD), Huntington’s disease, autism disorder, schizophrenia, MDD, etc. (Flores-Dorantes et al., 2020). Individuals with obesity have an earlier onset of AD which previously was considered an aging disease. Furthermore, obesity has been shown to exacerbate the development of memory dysfunction (Martin-Jiménez et al., 2017). Excessive sugar and refined grain intake have been found to increase amyloid accumulation in the lateral temporal lobe and posterior cingulate gyrus, which are classical pathological hallmarks of AD (Taylor et al., 2021).

Although multiple factors lead to obesity, the problem is largely on account of the increasing food energy supply. Studies have shown that the increase in food energy supply was higher compared to the model-predicted increases in energy intake, especially in high-income countries (Vandevijvere et al., 2015). This excessive food energy consumption is termed overnutrition. Interestingly, several human and rodent animal studies have proposed that cognitive impairment is the consequence of overfeeding instead of obesity. Maternal overnutrition detrimentally affects the learning and memory processes and neurochemical functions in second and third generations (Sarker and Peleg-Raibstein, 2018). A recent study of 92 healthy non-obese twin pairs demonstrated that a high-fat diet (HFD) damaged memory cognition (Schüler et al., 2018). Obesity and overnutrition can cause various neurobiological changes. It has been reported that brain atrophy was observed among obese individuals, especially in obese men. More specifically, gray matter volume (GMV) in the frontal and limbic brain areas was lower and obesity preferentially affected GMV over white matter volume (Dekkers et al., 2019). Furthermore, obesity significantly affected the hippopotamus, synaptic numbers, axon density, and the integrity of the blood-brain barrier (BBB).

As for the mechanisms of obesity or overnutrition inducing cognitive impairment, we will discuss the three main aspects in this review, including insulin resistance (IR), gut-brain axis, and neuroinflammation. Firstly, brain IR is proposed to be a common feature of metabolic and cognitive disorders (Kullmann et al., 2016). The gut microbiota has been recently shown to be a vital mechanistic link between unbalanced consumption and impaired cognition and has been termed the ‘gut-brain axis. Diet-induced obesity (DIO) or high nutrition independently impacts the composition and abundance of gut microorganisms (Cryan et al., 2019). Thirdly, microglia, relevant cytokines, and other immune factors also exert an indispensable and complex role. Importantly, it has been shown that cross-talk exists between insulin factors, gut metabolism factors, and neuroinflammation factors. Apart from the three aspects mentioned above, genetic related factors offer new perspectives to comprehend the mechanisms of diet or DIO-associated cognitive deficits (Flores-Dorantes et al., 2020). Most recently, studies have proved that knocking down microglia-specific genes, i.e., *Bmal1* could protect mice from HFD-induced obesity and improve memory (Wang X. L. et al., 2021). Anti-aging gene sirtuin 1 is possibly associated with the nature of the diet that targets the underlying mechanisms with relevance to diet- and obesity-induced cognitive impairment and neurodegenerative diseases. Sirtuin 1 is critical to the prevention of obesity, IR, cognition, and neurodegenerative diseases due to its role in metabolic activity, inflammation, and chronic diseases. It is manifested as a prominent part of the microbiota-gut-brain axis. Bacterial lipopolysaccharides (LPS) were shown to repress sirtuin 1 and then hinder mycotoxin metabolism, thus contributing to the accumulation of α-synuclein and Aβ and neurodegeneration (Martins, 2015). Human trials and animal experiments consistently found that the lack of sirtuin 1 aggravated IR. Sirtuin 1 and its transcriptional regulation of p53 were also linked with neuron glucose metabolism by influencing insulin receptors, implying its function in obesity- or diet-associated central IR (Kim et al., 2018; Ghadimi et al., 2021). Calorie restriction induces the expression of sirtuin 1 in mice and humans, while HFD triggers the loss of sirtuin1 (Chang and Guarente, 2014). The lack of sirtuin 1 decreases food intake and weight for energy balance. However, the inhibition of sirtuin 1 in some neurons augmented sensitivity to diet-induced obesity on account of decreasing energy expenditure (Herskovits and Guarente, 2014). As sirtuin is beneficial to cognition function, especially memory formation, its overexpression under chronic stress also yields negative effects. Hence, inhibiting sirtuin not only enhances the performance of the novel object location memory task but also reduces anhedonic behavior (Ferland et al., 2013). Therefore, sirtuin 1 is crucial in feeding responses and cognitive behaviors.

In conclusion, three multiple mechanisms may be interacting to culminate in cognitive dysfunction, but numerous knowledge gaps remain to be figured out.

## Cognitive Impairment in Obesity and Overnutrition

### Obesity or Overnutrition Induced Cognitive Dysfunction in Humans

Smith et al. (2011). analyzed data from different age groups, i.e., children and adolescents, adults to the elderly. They found that obesity and cognitive deficits were bidirectionally connected in the different age groups. Several reviews and meta-analyses have shown that obese individuals behave worse in tasks that examine executive function, verbal fluency, learning, and memory ability (Siervo et al., 2011; Fitzpatrick et al., 2013; Table 1). Higher BMI and older individuals have a lower executive function, memory performance, and processing speed due to lower GMV in corresponding brain regions. Specifically, memory decay has been associated with lower GMV in the frontal and thalamic BMI-associated regions (Kharabian Masouleh et al., 2016). However, studies on obese elderly individuals have shown their cognitive conditions to be complex. Some results claim that higher BMI in older individuals is not predictive of cognitive decline (Sturman et al., 2008). As for children and adolescents, recent studies have indicated that obesity is inversely associated with poorer cognitive competence such as executive function, attention, visuospatial skills, and motor skills, and may affect their academic achievements (Liang et al., 2014). Although a causal relationship between obesity and neurocognition is gradually being recognized, only a few studies have been performed. The study limitations are the narrow focus on executive function with a lack of verbal or memory tests for cognition measurement, as well as the overreliance on BMI as an indicator for obesity (Hartanto et al., 2019).

**TABLE 1:** The effect of diets and obesity on cognition in human.

References	Number of people, age, sex	Diet, duration	BMI (kg/m^2^)	Cognitive tests	Impact on cognitive impairments
Kivimäki et al. (2018)	1,349,857 participants	/	<20–>30	Clinical diagnosis of dementia	An inverse association between BMI and dementia. The hazard ratio of dementia per five-unit increase in BMI was 0.87.
Beyer et al. (2019)	748 participants 60–79 years old Male and female	/	17-42	VFT, TMT, CERAD word learning task	High body fat and visceral adiposity related with reduced gray matter volume and potentially reduced executive function in older adults.
Kharabian Masouleh et al. (2016)	617 participants 60-90 years old Male and female	/	17-41	TMT, semantic and phonemic verbal fluency and verbal memory	Higher BMI correlated with lower executive function. Age negatively correlated with memory performance and processing speed.
Cserjési et al. (2009)	30 participants ∼48.8 years old female	/	∼34.2	BDI, STAI, PANAS, DS, D2, TMT, SVFT, HSCT	Reduced mental flexibility and sustained attention capacity in obesity together with the presence of depressive mood.
Gonzales et al. (2010)	32 participants 40–60 years old Male and female	/	Normal: 18.5–24.9 Overweight: 25.0–29.9 Obese: ≥30	MMSE, WASI, CVLT-II and RCF delayed recall, COWA, BDI-II, STAI-T	The obese group displayed significantly lower task-related activation in the right parietal cortex.
Sánchez-SanSegundo et al. (2021)	87 participants 22–63 years old Male and female	/	≥25 kg/m^2^	CAB	Body fat was negatively associated with inhibition and working memory.
Yim et al. (2012)	67 participants 18–60 years old Male and female	/	Normal weight: 18.5–24.9 Overweight/obese: ≥25.0	NART-R; CVLT-II; TMT-A; TMT-B; CFQ; PDT; Shipley abstraction	BMI was negatively correlated with attention and psychomotor processing speed. Overweight and obese bipolar individuals had a significantly lower score on the verbal fluency test.
Olivo et al. (2019)	170,310 participants Male and female	/	Normal weight: 18.5–24.9 Overweight: 25–29.9 Obese: ≥30 kg/m^2^	FI, TMT, PMT, numeric memory test	Neuroticism led to greater weight variability over time, rather than to overweight/obesity itself.
Geda et al. (2013)	1,233 participants 70–92 years old Male and female	Reference group; moderate aloric intake group; High caloric intake group.	/	WMS; AVLT; TMT-B; BNT; WAIS.	High caloric intake related with MCI but not moderate caloric intake.
Smith et al. (2010)	124 participants Male and female	DASH	/	TMT B-A; Stroop Interference; Digit span; VFT; VPA; DSST; COWAT; Ruff 2 and 7 test.	Combining aerobic exercise with the DASH diet and caloric restriction improves neurocognitive function.
Yuan et al. (2022)	49,493 participants ∼48 years old Female	AMED, AHEI-2010, DASH	/	Seven-item questionnaire on memory and cognition changes	The odds of severe SCD were lower after improving diets for each SD higher in diet quality change, the reductions in risk were 11% for AMED, 5% for DASH, and 3% for AHEI-2010, respectively.
Kesse-Guyot et al. (2012)	6,850 participants 52.1 ± 4.6 years old Male and female	Healthy pattern Traditional pattern	/	RI-48 cued recall tests Verbal fluency tasks	Performance on verbal memory was positively associated with the level of adherence to healthy pattern.
Wu et al. (2019)	16,948 participants 45-74 years old Male and female	DASH, AHEI-2010, aMED, PDI, hPDI	/	SM-MMSE	The adherence to healthy dietary patterns in midlife is associated with a lower risk of cognitive impairment in late life in Chinese adults.
Eskelinen et al. (2008)	1,449 participants 65–80 years old Male and female	SFA, PUFA, MUFA from milk products and spreads	/	MMSE, LDST; immediate word recall tests; the Category Fluency Test; Purdue Peg Board task; Stroop test.	Abundant saturated fat (SFA) intake at midlife was associated with poorer global cognitive function, prospective memory and with an increased risk of MCI.
Kesse-Guyot et al. (2014)	2,983 participants Male and female	Carotenoid-rich dietary pattern (CDP)	/	VFT; TMT; RI-48 cued recall tests; Forward and backward digit spans.	CDP was positively associated with the composite cognitive performance score assessed 13 years later.

*TMT, trail-making test; CERAD, consortium to establish a registry for Alzheimer’s disease; VFT, verbal fluency tests; BDI, beck depression inventory II; STAI, state-trait anxiety inventory; PANAS, positive affectivity and negative affectivity schedule; DS, digit span memory test; SVFT, semantic verbal fluency test; D2, The D2 attention endurance test; TMT, the trail making test; HSCT, Hayling sentence completion task; BDI-II, beck depression inventory-II; COWAT, controlled oral word association test; CVLT-II, California verbal learning test-II; IQ, intelligence quotient; MMSE, mini mental state exam; RCF, Rey complex figure test; STAI-T, Spielberger trait anxiety inventor-T; WASI, Wechsler abbreviated scale of intelligence; CAB, CogniFit™ general cognitive assessment; NART-R, national adult reading test; CVLT-II, California verbal learning test; TMT-A, trail making test A; TMT-B, trail making test B; CFQ, cognitive failures questionnaire; PDT, process-dissociation task; FI, fluid intelligence; TMT, trail making test, PMT, pairs matching task; Reference group, 600–<1,526 kcals/day; Moderate caloric intake group, 1,526 to 2,143 kcals/day; High caloric intake group, >2,143 kcals per day; WMS, the Wechsler memory scale; AVLT, the auditory verbal learning test; BNT, Boston naming test; WAIS, the Wechsler adult intelligent scale; DASH, dietary approaches to stop hypertension; VPA, verbal paired associates; DSST, digit symbol substitution test; AMED, alternate mediterranean diet; AHEI-2010, alternate healthy eating index 2010; SCD, subjective cognitive decline; SM-MMSE, Singapore-modified version of the mini-mental state examination; aMED, alternate Mediterranean diet; PDI, overall plant-based diet index; hPDI, healthful plant-based diet index; MUFA, monounsaturated fatty acids; PUFA, polyunsaturated fatty acids; SFA, saturated fatty acids; LDST, letter digit substitution test; CDP, carotenoid-rich dietary pattern.*

Actually, besides the use of BMI as a marker of overall obesity, various parameters, including waist-to-hip ratio (WHR), waist circumference (WC), lipid accumulation products, and visceral adiposity index, are applied to identify abdominal obesity, an indispensable subgroup of obesity that also significantly influences the cerebral structure and cognitive performance (Wan et al., 2020). Studies found that higher WHR is independently linked with lower GMV volume in the network, which has a correlation with worse memory performance (Kharabian Masouleh et al., 2018). WHR and WC are also associated with slower processing speed, worse executive function, and declined linguistic fluency (Gunstad et al., 2010; Gardener et al., 2020). Comparatively, the limitation in using BMI as a measurement is that the abdominal and overall fat is hardly distinguished, as one hypothesis pointed out that central obesity was more clinically relevant to brain health (Gardener et al., 2020). Studies suggested that abdominal fat conferred an increased risk of dementia and cognitive impairment, while overall obesity might be protective (West and Haan, 2009). To be more specific, the influence of visceral adiposity (or intra-abdominal adiposity) rather than abdominal subcutaneous fat on CNS has been underscored in recent years, but experts’ opinions and experimental results have not reached a consensus yet (Smith, 2015). Some studies agreed on the deleterious impact of excessive visceral adiposity on the brain, suggesting that a high visceral adiposity index was associated with lower gray matter density in the caudal anterior cingulate cortex (Iceta et al., 2021). Moreover, elevated adiponectin mRNA expression in visceral adipose tissue (VAT) exhibited a positive correlation with the motor deficit, declined memory, and depression- and anxiety-like behavior (Metwally et al., 2019). Nevertheless, some studies examined VAT and gray and white matter, performed several cognition tests, and reported no significant relationship among them (Hsu et al., 2016). Further, some researchers found no association between visceral fat with dementia, but a close link between increased abdominal subcutaneous fat and decreased likelihood of dementia in women in late-life (Hsu et al., 2016). In summary, more studies are anticipated to compare the influence of visceral adiposity and abdominal subcutaneous fat on brain structure and function and identify potential reasons behind the difference.

Genetic and environmental factors give rise to the occurrence of obesity, and the latter involves insufficient exercise and an unbalanced diet. Western diet, which usually contains high fat, high sugar, and other high caloric nutrients, is negatively associated with cognitive function. From the disease-developing aspect, in a population-based case-control study, it is reported that high caloric intake was associated with a nearly two-fold increased odds of having an amnesic mild cognitive impairment (MCI) as compared to the reference group and amnesic MCI is likely to be a prodromal stage of AD (Geda et al., 2013). Additionally, autonomic nervous system (ANS) dysfunction including the accumulation of α-synuclein in PD is exacerbated by obesogenic diets and ameliorated by intermittent energy restriction (Griffioen et al., 2013). On the other side, higher adherence to a Mediterranean-type diet, which calls for fruit and vegetable intakes as well as replaces saturated fatty acid (SFA) with unsaturated fatty acid (UFA), was conducive to slower cognitive decline and the progression of MCI and AD (Bousquet et al., 2012).

From a behavioral aspect, data proved that abundant SFA intake from milk products in midlife brought about poorer global cognitive function and prospective memory (Eskelinen et al., 2008). From structural aspects, an overnutrition pattern diet also reduced the volume of hippopotamus (Jacka et al., 2015). From biological aspects, high nutrition diet is nowadays proposed as an inflammatory factor. Dietary inflammatory index (DII) is applied to assess, which associated with individuals’ inflammatory markers like C-reactive protein and IR (Shivappa et al., 2015; Saghafi-Asl et al., 2021).

Depression is also closely linked with obesity and diet patterns. With regard to obesity, it was found that obesity measured using various anthropometric indices was related to somatic-affective instead of cognitive-affective symptoms (Wiltink et al., 2013). The resulting metabolic and inflammatory dysfunction of obesity played a role in developing depression with an impact on the neuroimmune status and neural circuits controlling mood and emotional states (Fulton et al., 2022). Recently, some researchers combined this adverse metabolomics and inflammatory alterations with atypical, energy-related depressive symptoms and proposed an “immune-metabolic depression” (IMD) dimension (Alshehri et al., 2021). Moreover, the severity, age of onset, duration, and comorbidities of depression were associated with abdominal obesity, with a potential mechanism referring to neutrophil gelatinase−associated lipocalin (NGAL), a recently discovered adipokine (Marijnissen et al., 2014). Not only does obesity lead to a high risk of major depressive disorder (MDD), but also diet quality is independently related to depression or depressive symptomatology. Several randomized controlled trials proved that the Mediterranean diet could improve depressive symptoms as an adjunctive intervention and also reduce the incidence of the disease as beneficial prevention (Sánchez-Villegas et al., 2013; Parletta et al., 2019; Marx et al., 2021). However, these protective effects might fade away in older age if high adherence to a Mediterranean diet is not implemented (Psaltopoulou et al., 2013). Similarly, a small or traditional diet comprising fruit, vegetables, and fish positively influenced brain function and behavior (Francis et al., 2019). On the contrary, a western diet was significantly associated with psychiatric disorders (Jacka et al., 2010). The different effects were possibly due to different energy consumption in these dietary styles (Quirk et al., 2013; Rao et al., 2020; Yin et al., 2021). Furthermore, the underlying mechanisms underlying the effects of obesity and diet on depression and cognition are similar. The former involves inflammation, oxidative stress, epigenetics, mitochondrial dysfunction, gut microbiota, tryptophan–kynurenine metabolism, hypothalamic–pituitary–adrenal (HPA) axis, neurogenesis, and so on (Marx et al., 2021).

### Obesity or Overnutrition Induced Cognitive Dysfunction in Rodents

Studies have traditionally reckoned that excessive nutrition causes obesity and subsequent cognition impairments. Rats fed on a high saturated fat diet and sugar resulting in weight gain and body fat had difficulties in hippocampal-dependent serial feature-negative and hippocampal-independent simple discrimination problems (Davidson et al., 2012). Behavior tests have also been utilized to examine cognition, such as novel object recognition, attentional set-shifting task, Morris water maze, etc. in rats. Each of these entails the use of different brain areas for optimal performance. Rats in the obesity groups showed poor ability to perform complicated discrimination, interdimensional and extradimensional shift tasks (Bocarsly et al., 2015; Lewis et al., 2019; see Table 2). Multiple factors that influence obesity-related cognition deficits have been gradually revealed. It was found that depriving estrogen aggregated cognition and synaptic dysfunction. Furthermore, aging was found to play a role in diet-induced weight gain and the negative outcomes resulting from obesity (Pratchayasakul et al., 2015; Moser et al., 2019).

**TABLE 2:** The effect of diets and obesity on cognition in rodents.

References	Strain, sex, age	Diet, duration	Weight gain (g)	Cognitive tests	Impact on cognitive impairments
Sarker and Peleg-Raibstein (2018)	C57BL/6 mice Male and female 10 weeks	SC (4.5% fat); HFD (35% fat); 9 weeks	/	OFT; YMZT; PPI; two-way active avoidance learning.	Impaired cognition in PPI. Impaired learning and memory in the two-way active avoidance paradigm across three generations.
McNeilly et al. (2011)	Wistar rats Male ∼5 weeks	SC (7.4% fat); HFD (45% fat); 12 weeks	SC: ∼25–100; HFD: ∼25–175.	DMTP test; PR task; water maze task; OFT.	Performed worse in all aspects of an operant based delayed matching to position task fed with HFD. Not impaired spatial working memory in the open field water maze test.
Beilharz et al. (2014)	SD rats Male ∼8–10 weeks	Regular diet (59% carbohydrate, 15% fat); CAF S + diet (50% carbohydrate, 45% fat); S + diet (74% carbohydrate, 9% fat); CAF S- (49% carbohydrate, 47% fat); 5, 11, 20 days	∼75–175	Object and place recognition test.	Performed worse on the place, but not the object recognition task in rats fed by CAF S + diet, S + diet, CAF S- diet.
Stranahan et al. (2008)	SD rats Male 12 months	SC + plain water; HCD + 20% high-fructose corn syrup; 8 months	∼850	Water maze test.	Impaired spatial learning ability in rats fed by high-fat, high-glucose diet.
Pistell et al. (2010)	C57BL/6 mice, Male 12 months	SC; WD (41% fat); Corresponding low fat control diet; HFL (60% fat); 16 weeks/21 weeks	WD: 19.96 ± 1.08; C-WD: 6.42 ± 1.08; HFL: 55.28 ± 1.07; C-HFL: 42.2 ± 1.32.	Stone T-maze.	Not impaired cognition in the Stone T-maze in mice fed by WD. Impair cognition in HFL diets.
Lin et al. (2017)	Wistar rats Male 6 weeks	Control diet (9.5% fat); HFFD (37.5% fat, from soybean oil or coconut oil); 20 weeks	Control: 260.6 ± 2.87; Soybean Oil: 267.9 ± 2.05; Coconut Oil: 267.2 ± 2.54.	MWM task.	Impaired hippocampal-dependent spatial memory behavior after long-term high-fructose-high-coconut oil consumption
Sobesky et al. (2014)	Wistar rats Male 8 weeks	SC (17% fat); Medium-fat diet (42% fat); HFD (60.3% fat); 12 weeks	SC: ∼175; Med/HFD: 275;	CPE-FC paradigm.	Impaired memory in rats fed by HFD and effects was augmented with longer duration of HFD consumption
Davidson et al. (2012)	SD rats Male ∼7-8 weeks	SC (3.0 kcal/g); HED (4.5 kcal/g); 4 weeks	SC: ∼50; HED: ∼65;	FI, TMT, PMT, numeric memory test.	No significance on the simple discrimination Impaired throughout on the FN problem testing in HE-DIO groups
Bocarsly et al. (2015)	SD rats Male 10 weeks	SC (10% fat) HFD (45% fat) 8 weeks	HFD: ∼69-79.	AST; OFT; object memory test.	Worse performance in novel object recognition task and object-in-place task in obese rats. No difference in object location task
Yoshizaki et al. (2020)	C57BL/6J mice Male 7 weeks	SC (4.6% fat); HFD (60% fat); 7 weeks	SC: ∼3; HFD: ∼7.	OFT; YMZT; EPMT; sucrose consumption test.	Impaired cognition in OFT, YMZT. Impaired sucrose preference in the sucrose consumption test. Neither body weight nor body weight gain was associated with any of the behavioral traits we examined.
Spencer et al. (2017)	F344xBN F1 rats Male 3 and 24 months	SC (17% fat); HFD (60.3% fat); 3 days	SC: ∼0; Young HFD: ∼2; Aged HFD: ∼4.	MWM; contextual fear-conditioning.	Impaired long-term, but not short-term contextual and auditory-cued fear memory Partially impaired spatial memory in aged rats
Beilharz et al. (2016a)	SD rats Male ∼6 weeks	SC (21% fat, 16% sugar); Sugar diet (19% fat 30% sugar); SFA (47.5% fat, 11% sugar); PUFA (45% fat, 11% sugar); 8 days, 12–13 days	Control: 319.7 ± 5.30; Sugar: 335.8 ± 5.50; SFA: 332.1 ± 3.50; PUFA: 319.3 ± 4.10;	Object and place recognition test.	Impaired on hippocampal dependent place recognition memory in rats consuming SFA and Sugar
Beilharz et al. (2016b)	SD rats Male ∼6 weeks	SC (plain water); Sugar diet (plain water + 10% sucrose solution); Caf + Sugar diet (plain water + 10% sucrose solution); 1, 5, 8 days	SC: ∼40; Sugar: ∼40; Caf + Sugar: ∼65.	Object and place recognition test.	Impaired selective hippocampal-dependent memory deficits Results of Sugar diet and Caf + Sugar diet are similar
Denver et al. (2018)	C57BL/6 mice Male 7–14 weeks	SC (10% fat); HFD (45% fat); 18 days, 34 days, 10 weeks, 21 weeks	SC: ∼5; HFD: ∼15.	NORT; MWM.	Consistently impaired Recognition memory in HFD-fed mice Impaired spatial learning in 18-day and 21-week HFD-fed mice
Beilharz et al. (2018)	SD rats Male ∼6 weeks	SC (14% fat); Cafeteria diet (plain water + 10% sucrose solution) 11 and 19 days	∼20–26	Place recognition task; object recognition task; EPMT.	Impaired hippocampal-dependent cognitive deficits on the place task Impaired memory deficits on the perirhinal-dependent object task

*PPI, prepulse inhibition; SC, standard chow; HFD, high-fat diet; PR, progressive ratio; CAF S + diet, cafeteria diet; S + diet, sugar diet; CAF S- diet, modified cafeteria diet; SD, Sprague–Dawley; HCD, high-calorie diet; WD, western diet; HFL, high-fat lard diet; MWM, Morris water maze; HFFD, high-fructose-high-fat diets; CPE-FC, contextual pre-exposure fear-conditioning; HED, high-energy diet; DIO, diet-induced obesity; AST, attentional set-shifting task; OPT, open field test; EPMT, elevated plus maze test; YMZT, Y-maze test; SFA, saturated fatty acid; PUFA, polyunsaturated fatty acid; NORT, novel object recognition test.*

However, conflicting results regarding the relation between high nutrition and cognitive impairment have also been reported. Some studies have demonstrated that excessive dietary fat for long periods does not induce learning deficits or spatial memory deficits, although HFD-induced obesity may have a detrimental influence on cognitive flexibility (Leyh et al., 2021).

Could high nutrition affect cognitive behavior? Recent studies have investigated the influence of obesity and high nutrition and suggested that overnutrition alone could affect cognition. Sobesky et al. (2014) found that mice fed on an HFD performed worse in contextual pre-exposure fear conditioning, which reflects memory function. Interestingly, when the HFD diet was reversed, the negative impact on memory was alleviated. This demonstrated that a high nutrient diet, not weight gain mediated the negative effects on memory (Sobesky et al., 2014). Additionally, studies have found that HFD mice demonstrated hyper-locomotion in the open-field test and Y-maze test, and impaired sucrose preference in the sucrose consumption test. These behavioral traits above were not associated with body weight or body weight gain (Yoshizaki et al., 2020). In conclusion, the above studies demonstrated that obesity was not a necessary factor that contributed to cognitive impairment induced by an excessive nutritional diet.

High nutrition-induced cognition decline and relevant brain structural alterations mainly affect the hippopotamus. Short-term high fat and sugar exposure or high sugar diet alone induced hippocampal-dependent place recognition memory before weight changes and were the result of hippocampal inflammation and oxidative stress (Beilharz et al., 2014). High fat and high refined sugar diet for 2 months were shown to be detrimental to spatial learning performance with reduced hippocampal levels of brain-derived neurotrophic factor (BDNF) (Molteni et al., 2002). In addition to a high fat, high-fructose-high-coconut oil consumption significantly affected the hippocampal-dependent spatial memory behavior determined using the Morris Water Maze tasks. This was closely related to reduced expression levels of leptin protein, as well as mRNA expression levels of leptin receptor and SCD1 mRNA in the hippocampus (Lin et al., 2017).

The negative effects of a high nutritional diet were not limited to one generation. Maternal HFD exposure brought long-term and detrimental effects on cognition across three subsequent generations. Impaired cognition was most evident in prepulse inhibition and the two-way active avoidance paradigm. Furthermore, the observed neurochemical changes like reduced dopamine, glutamate, and gamma-aminobutyric acid (GABA) levels suggest altered dopaminergic, glutamatergic, and GABAergic signaling. This may provide some explanation for the behavior of children from mothers consuming HFD (Sarker and Peleg-Raibstein, 2018).

## Overnutrition or Obesity-Induced Cognitive Impairment Related to Brain Imaging

Multiple studies have demonstrated the negative impacts of weight gain and high-nutrient food intake on the CNS. Obesity leads to dysregulation of protein degradation in the hippocampus that correlates with spatial memory. High free fatty acid (FFA) and triglycerides impair the long-term balance of *N*-methyl-*d*-aspartate in the hippocampus to mediate cognitive deficits (Farr et al., 2008; McFadden et al., 2020). With the advancement of imaging technologies, alterations in brain structure and glial cells and neurons could be investigated in individuals with overnutrition and obesity.

### Changes in Brain Volume and Blood-Brain Barrier Permeability

With regards to the brain, the reduction of gray matter, compromised white matter integrity, and BBB hyperpermeability are well-known effects induced by obesity. Higher BMI is associated with lower GMV or cortical thickness within similar areas of the frontal lobes, cingulate gyrus, amygdala, dorsal striatum, thalamus, cerebellum, and affects children, adolescents, and adults (Kharabian Masouleh et al., 2016; Westwater et al., 2019; Laurent et al., 2020). The data above was based on magnetic resonance imaging (MRI), which was able to discriminate gray and white hypothalamic structures and highlight finer structures like brain nuclei (Baroncini et al., 2012). Interestingly, the volume of subcortical gray matter structures in men appears to be more affected by the total body fat (TBF) ratio compared to women. This is probably due to the positive effect of estrogen on the MetS (Mauvais-Jarvis, 2015). Apart from the volume and thickness of gray matter, studies using diffusion tensor imaging (DTI) to create apparent diffusion coefficient (ADC) maps of gray matter, have found that gray matter ADC in the left and right amygdala and the right parietal region was significantly and positively associated with higher fibrinogen levels among overweight/obese individuals (Cazettes et al., 2011). Moreover, lower gray matter density in the caudal anterior cingulate cortex was strongly associated with a high visceral adiposity index and metabolic alterations (Iceta et al., 2021).

As for white matter integrity, global and tract-specific white matter integrity were measured using fractional anisotropy and mean diffusivity in DTI. Hyperintensity of white matter has been shown to have better local network efficiency, information processing speed, and cognitive function (Vergoossen et al., 2021). It has been observed that obesity is related to higher coherence but the lower magnitude of white matter microstructures and higher TBF were related to reduced commissural fibers, association fibers, and projection fibers (Dekkers et al., 2019). Hyperpermeability of BBB in obese individuals has been observed and accounts for increased albumin content in the hippocampal, reduced claudin-5 protein levels, and collagen IV immunostaining, which eventually results in the transmigration of peripheral immune cells (de Aquino et al., 2018). Since BBB leakage measured by dynamic contrast-enhanced magnetic resonance imaging (DCE-MRI) is a potential biomarker for bipolar disorder and dementia (Kamintsky et al., 2020; Chagnot et al., 2021), we believe that BBB integrity will be a useful indicator of obesity-related cognitive impairments in the future. Additionally, concerning brain functional connectivity measured using functional magnetic resonance imaging (fMRI), it was observed that global brain connectivity (GBC) was altered in obesity, which included reduced GBC in prefrontal and feeding circuits and increased GBC in the dorsal attention network (Geha et al., 2017).

In conclusion, MRI and other methods clearly showed negative changes in the gray matter, white matter, BBB, and functional connectivity in the brain under DIO and hence demonstrated a structural foundation for cognitive dysfunction and behavioral abnormality.

### Changes in Brain Microstructure

Under short-term HFD exposure before the obvious weight gain, reactive gliosis was observed in the hypothalamic arcuate nucleus (ARC) of rats and mouse models. Although T2-weighted imaging (T2WI) to study the effects of obesity and HFDs on brain tissue is rare, the results demonstrated that T2 hyperintensity was associated with the presence of gliosis in different pathologies (Lizarbe et al., 2020). It has been observed that microglia numbers were increased, enlarged, and transformed to a more activated morphology, as well as astrocytes forming a dense fibrous network. Furthermore, by examining gliotic changes below the visual detection threshold, clinical studies have demonstrated similar results that obese individuals have associated gliosis in the mediobasal hypothalamus (MBH) (Thaler et al., 2012). Negative effects on the synapse have been shown to be accompanied by microglial activation. Studies have demonstrated that in dietary obese mice, activated microglia induced a series of synaptic alterations including the impairment of hippocampal synaptic plasticity, reduction of dendritic spine density and sites of excitatory synapses, and promoted synaptic stripping (Hao et al., 2016; Cope et al., 2018). A negative correlation between BMI and synaptic density was also observed by [11C] UCB-J positron emission tomography (PET) imaging. In this approach, synaptic vesicle density was measured through synaptic vesicle glycoprotein 2A (SV2A), a transmembrane glycoprotein ubiquitously and homogeneously expressed in presynaptic vesicles, while PET was used to measure [11C] UCB-J binding to SV2A (Asch et al., 2022).

Concerning neuronal responses to diet or obesity, DIO and HFD consumption induced injury and loss of Pro-opiomelanocortin (POMC) neurons in the ARC and Sim1 neurons of the paraventricular nucleus (PVN). While the former was more specific to male mice, the latter shared features of exposure to HFD for both genders. POMC acts as an agonist for neurons expressing the type IV melanocortin receptor (MC4R) to increase energy expenditure and reduce food intake. Thus, POMC injury accelerated the progression of DIO (Iqbal et al., 2021). Studies have demonstrated that inhibition of POMC neurons by melanin-concentrating hormone (MCH) through the SIRT1/FoxO1 pathway resulted in hyperphagia and obesity (Al-Massadi et al., 2019). In addition to imaging techniques to study neuronal impairment like POMC, new methods are being or have been developed. For example, Lactam bridge-cyclized α-melanocyte-stimulating hormone analogs have been investigated for their suitability for MC1R-targeted imaging, especially for single-photon emission computed tomography (SPECT) (Zhang et al., 2017). Dopamine plays an important role in eating behavior and is one of the main reasons for obesity. Measured using dopaminergic PET and SPECT, reduced dopamine signaling in individuals with elevated BMI has been observed to be consistent with reduced dopamine synthesis capacity in the dorsal striatum (Val-Laillet et al., 2015). PET is also able to reflect brain substrate metabolism, albeit relatively limited and technically different from PET imaging of human diabetes and obesity (Guzzardi and Iozzo, 2019).

Neuroimaging technologies such as PET, SPECT, fMRI, and pharmacogenetic fMRI have been useful for identifying obesity-related abnormalities in a wide range of brain areas in obese and high nutritional subjects. This provides advantages for scientific studies, clinical diagnosis, and targeted treatments.

## The Role of Central Insulin Resistance in Obesity- or Overnutrition-Associated Cognitive Impairment

### Regulation of Metabolism and Neural Function by Insulin

It is well-known that insulin, a peptide hormone secreted by pancreatic β-cells, regulates glucose metabolism in peripheral tissues. Regarding the central source of insulin, peripheral insulin enters the brain *via* an insulin-mediated pathway and triggers two distinct signaling cascades, namely the phosphoinositide 3-kinase (PI3) pathway and the mitogen-activated protein kinase (MAPK) pathway, which is crucially involved in insulin sensitivity (Begg, 2015; Kullmann et al., 2020a). However, whether the brain locally synthesizes insulin is unclear. Previous studies suggested the presence of immunoreactive insulin in the brains of humans and rodents, which was opposed by subsequent studies believing that “little or no insulin is produced in the CNS” (Banks, 2004; Blázquez et al., 2014). Recently, emerging evidence showed that insulin was produced by a subpopulation of inhibitory neurons of the cerebral cortex to rapidly regulate the energy homeostasis of the neural network (Csajbók and Tamás, 2016). Overall, the source of insulin in the brain entails further exploration to draw a definite conclusion. Moreover, a systematic review of patients with T2DM suggested that improving glycemic control by peripheral insulin affects brain function (Geijselaers et al., 2015). In addition, other studies have proved that the brain inherently contains insulin, which is involved in metabolic modulation and neural circuit function.

Insulin regulates peripheral metabolism, including regulation of appetite, white fat mass, hepatic glucose output, etc. The influence of CNS insulin on decreasing dietary consumption and body weight is one of the earliest discoveries on the topic. For instance, researchers reported that intracerebroventricular (ICV) injection of insulin could inhibit food intake, which is partly regulated by unexcited orexigenic agouti-related peptide (AgRP) neurons and activated ATP-dependent potassium channels in hypothalamic neurons (Könner et al., 2007; Mc Allister et al., 2015). Similarly, Koch et al. (2008) found that chronic ICV insulin treatment promotes fat mass, fat cell size, and adipose tissue lipoprotein lipase expression.

**FIGURE 1:**
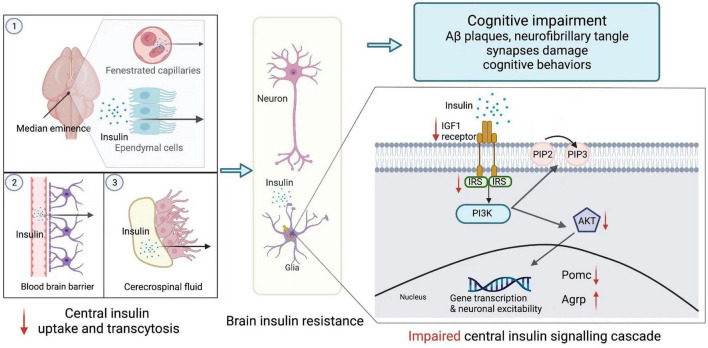
Brain insulin resistance. Peripheric insulin reaches its targets in the brain mainly through three approaches: **(1)** passive extravasation of the fenestrated capillaries and ependymal cells of the median eminence; **(2)** transcytosis from the blood-brain barrier; **(3)** tanycyte-mediated transport from cerebrospinal fluid. However, under the overnutrition condition, brain insulin transportation is declined. In the CNS, insulin exerts its effects *via* central insulin signaling cascade. Insulin binds to insulin-like growth factor 1 (IGF1) or insulin receptors (INSR) expressed on the endothelial cells. After receptors recognition, the phosphoinositide 3-kinase (PI3K)/protein kinase B (Akt) pathway is activated. PI3K converts phosphatidylinositol (3,4)-bisphosphate (PIP2) into phosphatidylinositol (3,4,5)-trisphosphate (PIP3), while Akt promotes the expression of pro-opiomelanocortin (POMC) and represses the expression of agouti-related peptide (AgRP) within the neuron. This brain insulin signaling is impaired during central insulin resistance, which is appeared to be reduced phosphorylation of insulin receptors, insulin receptor substrates, and Akt. AgRP, agouti-related peptide; Akt, protein kinase B; IGF1, insulin-like growth factor; INSR, insulin receptors; PI3K, phosphoinositide 3-kinase; PIP2, phosphatidylinositol (3,4)-bisphosphate; PIP3, phosphatidylinositol (3,4,5)-trisphosphate; POMC, pro-opiomelanocortin.

Besides, neurons express insulin receptors to maintain synapse density, dendritic plasticity, and normal functions (Chiu et al., 2008). Insulin receptor signaling promotes dendritic spine establishment and excitatory synapse development through activating PI3K/Akt/mTOR and Rac1 signaling pathways (Lee et al., 2011). Insulin signaling also benefits cognitive competence. Insulin/IGF-1 signaling charges spatial learning and memory in the hippocampus (Soto et al., 2019). In contrast, serine/threonine-protein kinase phosphorylation, a marker of IR is correlated with a lower level of global cognitive function (Arvanitakis et al., 2020). Furthermore, central insulin is involved in AD and other psychotic disorders. For AD patients, insulin signaling ameliorates amyloid-β (Aβ) fibrils and tau protein hyperphosphorylation. Claxton et al. (2015) found that intranasally administered insulin augments their verbal and visuospatial working memory (see Figure 1).

### Central Insulin Resistance and Cognition

Insulin resistance refers to the impaired ability of insulin to exert its influence on target tissues (Kullmann et al., 2016). In the periphery, systematic IR is a hallmark feature of obesity. However, it has to be pointed out that the brain is an insulin-sensitive organ, and IR has been observed in the CNS of individuals with obesity and metabolic diseases like T2DM.

Recent advances in neuroimaging techniques have further promoted studies on brain IR in obese people. For example, a study on magnetoencephalography first reported brain insensitivity to exogenous insulin in obese adults in 2006. Tschritter et al. (2006) found that insulin infusion stimulates spontaneous cortical activity in healthy subjects, which was not observed in obese subjects. Moreover, Pegueroles et al. (2020) found a higher BMI augmented brain glucose metabolism and reduced cortical thickness after performing imaging brain glucose metabolism using fluorine-labeled fluorodeoxyglucose ([18F]-FDG) PET. Apart from obesity, visceral fat or body fat distribution is also linked with central insulin sensitivity (Kullmann et al., 2020b). This is achieved by an increase in functional connectivity between the dorsal medial prefrontal cortex and hippocampus after insulin administration (Kullmann et al., 2017). Therefore, central insulin sensitivity is vital to prevent visceral fat accumulation, which not only causes white matter lesions but also increases the risk of developing dementia (Ozato et al., 2021).

Obesity has long been known as a product of nutritional excess. However, independent of obesity, overnutrition can directly blunt central insulin sensitivity, even before peripheral insulin signaling is impaired. After short-term HFD exposure, rats were found to be unresponsive to 3rd-cerebral ventricle insulin administration, which is embodied by decreased ability to reduce food intake and activate hypothalamic signal transduction (Posey et al., 2009; Clegg et al., 2011). Gray and colleagues found that HFD feeding diminishes brain endothelial cell insulin uptake and transcytosis (Gray et al., 2017). Yet, whether brain insulin delivery is affected by IR needs to be further explored. Some studies have reported reduced insulin transportation into the cerebrospinal fluid (CSF) in several IR states (Heni et al., 2014). The underlying mechanisms of overnutrition leading to central IR have also been unveiled. Zhang et al. (2008) delivered IKKβ*^rm CA^* bilaterally into the MBH of C57BL/6 mice and found that lIKKβ/NF-κB interrupts central insulin signaling in the hypothalamus because IKKβ*^rm CA^* decreases insulin-stimulated Akt activation and PIP production and hinders tyrosine phosphorylation in insulin receptor and insulin receptor substrate 2 (IRS2).

Previous studies explored the relationship between cognition diseases and IR. An 11-year follow-up study observed that IR predicted poorer verbal fluency and a steeper decline in verbal fluency. It indicated that IR might serve as a precursor to cognitive disorders and intranasal insulin might become a potential therapy to hinder cognitive function decline (Kullmann et al., 2016; Ekblad et al., 2017). At present, the influence of central insulin on neuropathological characteristics of AD has been widely studied. High levels of pSer312-IRS-1 (ineffective insulin signaling) and lower levels of p-panTyr-IRS-1 (effective insulin signaling) are the exosomal biomarkers of brain IR. Studies proved that these biomarkers were associated with brain atrophy in AD (Mullins et al., 2017). Besides, in the cerebellar cortex of patients with AD and without T2DM, the response to insulin signaling in the IR→IRS-1→PI3K signaling pathway is markedly reduced. Moreover, increased biomarkers of brain IR are positively associated with aggregation of oligomeric Aβ plaques and dysfunction of working memory, which demonstrates that brain IR is highly related to cognition decline (Talbot et al., 2012).

Knowing that brain IR acts as a shared pathological feature of metabolic dysfunction and cognitive disturbances and that overnutrition and obesity both affect cognition, brain IR may be a bridge in the influence of nutritional excess and obesity on cognitive function. Nowadays, multiple studies combined factors of HFD, brain IR, and cognition impairment and found that HFD and a high-fat/high-sucrose (HFS) diet increases telencephalic IR [increased expression of IR marker IRS1-pS (616)], as well as amyloid-beta deposition and neurofibrillary tangle formation, having a deleterious effect on synaptic integrity and cognitive behavior (Arnold et al., 2014; Kothari et al., 2017). However, few of these studies truly illustrated underlying mechanisms and casual relationships, which should be addressed by future research.

## The Role of the Gut-Brain Axis in Obesity- or Overnutrition-Associated Cognitive Impairment

Gastrointestinal function exerts an influence on CNS and vice versa. Recently, increasing studies have focused on investigating bidirectional gut-to-brain communication as one of the mechanisms in some cognitive disorders. Over the years, a gut-brain axis has gradually become a novel therapeutic target for mental and cognitive-related diseases. In this part, we discussed how overnutrition and obesity influence the density and composition of the gut microbiome and the mechanisms through which the gut-brain axis influences cognitive function.

There is a hierarchic four-level neural network between the gastrointestinal system and CNS, which respectively refers to the enteric nervous system (ENS) represented by neurons of the myenteric and submucosal plexi and enteric glial cells, the prevertebral ganglia monitoring peripheral visceral reflex responses, the ANS within the spinal cord and lastly higher brain centers (Mulak and Bonaz, 2015). Holmqvist et al. (2014) suggested that α -synuclein-enriched Lewy bodies, a cellular hallmark of PD, propagate from the gut to the brain *via* the vagal nerve in rats. However, the relationship between gut and brain is not only associated with neurodegeneration like AD or PD but also related to metabolic balance and food intake behaviors. As we know, the vagal is a major afferent nerve in gut-brain communication. Among various kinds of the vagal, glucagon-like peptide-1 receptor (GLP1R)-expressing vagal afferent induces satiation through gut-derived anorexigenic signals. Independent of food intake, GLP1R vagal afferent also improves glucose tolerance and lowers blood glucose levels. In contrast, GPR65-expressing vagal afferent stimulation increases blood glucose (Borgmann et al., 2021).

**FIGURE 2:**
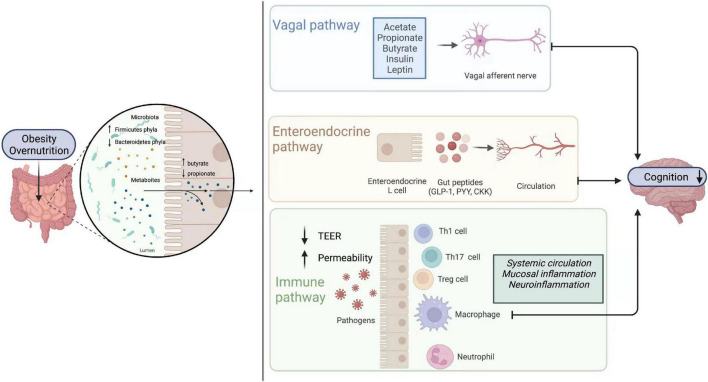
Gut-brain axis. Obesity or high nutrition diets lead to alterations of gut microbiome composition. There is an increase in Firmicutes phyla and the metabolites (butyrate) they yield, while Bacteroidetes phyla and their productions (propionate) decrease. The change of the enteric microbiome influences brain function directly or indirectly through vagal, endocrine, and immune pathways. Metabolites directly activate vagal afferents and transfer signaling to the brain in the vagal pathways. In the endocrine pathway, enteroendocrine L cells release gut hormones such as glucagon-like peptide 1 (GLP1), peptide YY (PYY), cholecystokinin (CCK), and so on, which can in turn influence learning, memory and mood. In the immune pathway, immune cells like T helper 1 (Th1), T helper 17 (Th17), regulatory T cell (Treg), neutrophils, and macrophages are stimulated. Additionally, intestinal mucosal permeability and transepithelial electrical resistance (TEER) are altered. CCK, cholecystokinin; GLP1, glucagonlike peptide 1; PYY, peptide YY; TEER, transepithelial electrical resistance; Th1, T helper 1; Th17, T helper 17; Treg, regulatory T cell.

However, the past 15 years have witnessed the emergence of the microbiota as one of the key regulators of gut-brain function. Accordingly, a novel concept, i.e., “microbiota-gut-brain axis” has been proposed (Figure 2).

### Bidirectional Communication Between the Brain and Enteric Microbiome

The brain can modulate functions and environments of the gut, which then influence enteric microbiota *via* a series of parallel outflow systems, including sympathetic and parasympathetic branches of the ANS, the HPA axis, and endogenous pain-modulation systems. For example, neuronal and neuroendocrine signaling like norepinephrine released in the intestine under stress or trauma increases the virulence of Campylobacter (Cogan et al., 2007).

At the same time, the gut microbiome produces various signaling molecules which connect CNS through endocrine, immune, and neural signaling mechanisms (Rhee et al., 2009). For the endocrine aspect, enterochromaffin cells work as signal transducers. These cells promote CaV-dependent serotonin release and form synaptic-like structures with 5-HT3R-expressing nerve fibers for transmitting signals (Bellono et al., 2017). Additionally, tryptophan catabolism produced by *Edwardsiella tarda* and other gut microbiome stimulates enteroendocrine cells, which activates vagal sensory ganglia and secretes 5-HT (Ye et al., 2021). As for immune pathways, the colony of diverse bacterial communities in the gut induces IL-17-producing γδ T cells that are transferred to the brain under a specific circumstance like an ischaemic injury, where they exacerbate inflammation and tissue impairment (Powell et al., 2017). At last, although the gut microbiota communicates with the brain *via* endocrine and immune pathways, vagus nerve signaling is probably the fastest and most direct approach (Fülling et al., 2019). Goehler et al. (2005) found that live *Campylobacter jejuni* activates c-Fos in neurons in the vagal sensory ganglia and the solitary tract nucleus in mice. Moreover, Bravo et al. (2011) suggested that *Lactobacillus rhamnosus* reduces GABA (Aα2) mRNA expression in the prefrontal cortex and amygdala, which were not found in vagotomized mice. Similarly, *Limosilactobacillus reuteri* improves social interaction-induced synaptic plasticity in a vagus nerve-dependent manner (Sgritta et al., 2019).

### Gut Microbiota in Obesity

In both the human and mouse models of obesity, the most acknowledged alteration in the gut microbiome is an increase in the relative abundance of Firmicutes and a decrease in Bacteroidetes compared with lean controls (Leigh and Morris, 2020). Clinical studies suggested that obese participants with more marked adiposity and increased fat mass are more likely to have low bacterial genes in the gut (<480,000 genes) (Le Chatelier et al., 2013). Also, higher BMI has been associated with lower alpha diversity, species richness, and beta diversity (Geisler et al., 2021).

Based on that, pertinent therapeutic methods targeting the gut-brain axis have gradually emerged. Erchen Decoction helps with obesity and lipid metabolism disorder by adjusting gut microbiota’s structure, abundance, and function following reduced metabolite -propionic acid (Zhao et al., 2021). Ussar et al. (2015) suggested that transplanting microbiota from obesity-resistant mice to germ-free mice with obesity susceptibility can ameliorate their specific metabolic consequences of obesity. Also, Nicolucci et al. (2017) found that prebiotics decreases body weight z-score, percent body fat, percent trunk fat, and serum level of IL-6 in children with overweight or obesity *via* selectively adjusting intestinal microbiota.

### Gut Microbiota in Overnutrition Conditions

Clinical studies pointed out that gut microbiota could rapidly respond to an altered diet. Compared to a plant-based diet, a greater change in gut microbial abundance was found in an animal-based diet with high fat and high protein (David et al., 2014). Similar results were also observed in animal experiments. HFD increased the abundance of Firmicutes in different strains of mice. Among these operational taxonomic units (OTUs), *Clostridiales*, *Roseburia intestinalis*, and *Ruminococcaceae* were positively correlated with specific metabolic phenotypes (Ussar et al., 2015).

Interestingly, weight gain is not a necessary factor contributing to cognitive impairment induced by a high nutrition diet. For example, Ravussin et al. (2012) found no difference in weight between mice fed with a high-fat diet and mice fed with a relatively low-fat diet; yet, the abundance of OTUs belonging to the *Lachnospiraceae* and the *Deferribacteraceae* was different, which also explained different circulating leptin and inflammation markers between them. Hildebrandt et al. (2009) found a decrease in Bacteroidetes and an increase in both Firmicutes and Proteobacteria in obese mice or RELMbeta knockout mice who remained comparatively lean when fed with HFD. Maternal HFD, but not maternal obesity, reconstructed the offspring’s intestinal microbiome (Liu et al., 2021). However, a recent study pointed out that refined HFD promotes the alpha diversity in the cecum and colon and decreases the Firmicutes: Bacteroidetes ratio. This is inconsistent with previous studies, which is probably because of the higher content of fiber in refined HFD (Wang et al., 2020).

Gradually, surging evidence in animal studies revealed that interventions affecting the enteric microbiome and targeting specific bacterial species could influence cognition. The alteration of the gut microbiome prevented, ameliorated, and even restored brain function after obesity or diet-induced cognitive deficits. It also benefited cognitive performance in healthy models. In the future, similar microbiome interventions are expected to apply in clinical treatment as well as benefit patients suffering from cognition degeneration.

## The Role of Neuroinflammation in Obesity- or Overnutrition-Associated Cognitive Impairment

For healthy individuals, the peripheral immune cells and cytokines are restricted by BBB and the blood-to-CSF barrier. However, in some neuroinflammatory diseases, several tight junction proteins of the barriers are disrupted and hence the leukocytes and T cells migrate across multiple pathways to reach brain parenchyma. In the CNS, CD4^+^ Th17 and Th1 lymphocytes produce IL-17 and IFN-γ, respectively, to engage in axonal damage and oligodendrocyte death (Liebner et al., 2018). Importantly, it has been proved that obesity or an overnutrition diet has an individual correlation with the leakage of BBB, leading to the development of neuroinflammation and cognitive dysfunctions (Guillemot-Legris and Muccioli, 2017; Więckowska-Gacek et al., 2021). The definition of neuroinflammation hasn’t reached a clear consensus. Generally speaking, CNS is endowed with an elaborated response repertoire termed “neuroinflammation,” which enables it to cope with pathogens, toxins, traumata, and degeneration (Xanthos and Sandkühler, 2014).

### Obesity or Overnutrition Induced Neuroinflammation

Diet-induced obesity propels neuroinflammation, as proved that DIO-induced morphological changes in the hypothalamic microglia and macrophages were similar to those of reactive microglia induced by peripheral nerve injury (Kälin et al., 2015). Interleukin plays a key role in obesity while associated with neuroinflammation. In the ARC of the hypothalamus of adult mice, IL6 and its canonical receptor are detected, and elevated expression of IL6 protects mice from DIO (Bobbo et al., 2021). Most recently, scientists figured out IL-27-IL-27Rα signaling in protecting against DIO and ameliorating IR (Wang Q. et al., 2021) but whether IL-27 is associated with inflammation alleviation in CNS as the base of common characteristics of obesity and neuroinflammation still remains further explore.

If we intend to link adiposity and cognitive disorders from an immune perspective, HIV-infected patients with immune defects would be a special group to explore. HIV directly or indirectly targets white adipose tissue, thus contributing to systematic inflammation or inflammation of distant organs such as the brain through adipokine secretion and lipotoxicity (Bourgeois et al., 2021). Neuroinflammation (choline and myoinositol) was detected in patients diagnosed with HIV-associated neurocognitive disorders (HAND), which was accompanied by less cortical gray matter and more abnormal white matter. It explained the comorbidity of acquired immune deficiency syndrome (AIDS) and cognitive decline to some extent (Saloner et al., 2019). Moreover, central obesity-related to AIDS predicts a higher prevalence of neurocognitive impairment, appearing as a decline in motor function, learning, and memory (McCutchan et al., 2012; Rubin et al., 2019). Therefore, it is possible that adiposity, especially central obesity, induces systematic and neuronal inflammation and causes cognitive deficits.

**FIGURE 3:**
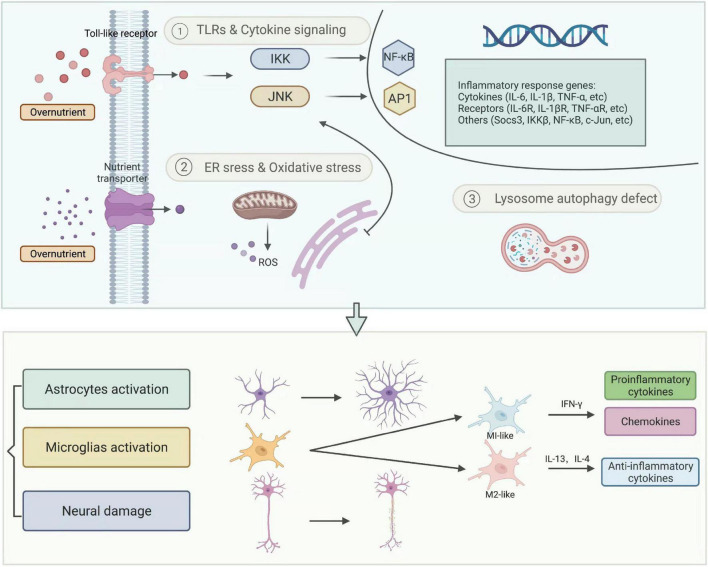
Neuroinflammation. Diet, lifestyle, and other external factors cause chronic overnutrition, which leads to IKK/NF-κB and JNK/AP1 directed inflammatory response and other intracellular organelle stresses, such as endoplasmic reticulum (ER) stress, oxidative stress, and lysosome autophagy defect. These cellular and molecular reactions eventually result in neuroinflammation, represented by activated astrocytes, microglia, and damaged neurons. AP1, activator protein-1; IKK, IκB kinases; JNK, c-jun *N*-terminal kinase; NF-κB, nuclear factor kappa-B; ROS, reactive oxygen species.

However, recent studies proved that overnutrition might cause inflammatory responses in the CNS, which appear earlier than the onset of obesity. Surprisingly, even after 1-day HFD exposure, increased microglial reactivity and pro-inflammatory cytokines like TNF have been observed in the MBH in mice and rats (Thaler et al., 2012). Diet-induced neuroinflammation is a key factor associated with obesity. It acts by regulating food intake behavior as well as microglial activation, thus consequently altering hypothalamic neurons at the earliest stage of obesity (see Figure 3).

A great amount of experimental data show strong bonds between overnutrition-induced cognitive impairment and hypothalamic or extra-hypothalamic inflammation. Hsu et al. (2015) found that a high fructose corn syrup-55 intake damage hippocampal-dependent spatial learning and memory in adolescent rats, which is probably associated with elevated expression of pro-inflammatory cytokines (IL6, IL1β) in the dorsal hippocampus. Moreover, Spencer et al. (2017) found that short-term exposure to HFD impaired hippocampal-dependent and amygdala-dependent memory in aged rats, accompanied by elevated IL-1β protein. When IL-1RA was centrally administrated, previous memorial deficits were alleviated, which indicated that IL-1β related neuroinflammation has a role in cognitive damage (Spencer et al., 2017). Interestingly HFD does not affect the basal expression of pro-inflammatory cytokines at the periphery or in the brain but enhances the expression of IL-1β and TNF-α, specifically in the hippocampus (Boitard et al., 2014).

Overfeeding mice with an SFAs diet caused an accumulation of microglia in the MBH and activated to M1 phenotype, followed by increased hypothalamic SFA levels (Beilharz et al., 2014). Moreover, a cascade of inflammatory signaling triggered by SFAs has been discovered. After combining with TLR4 and then activating myeloid differentiation primary response gene 88 (MyD88), its downstream signaling, including IKK complex and NF-κB, was triggered, which eventually led to the expression of proinflammatory genes in the hypothalamus (Milanski et al., 2009). Except for TLR/NF-κB signaling, IKKβ/NF-κB signaling, a master switch and main monitor of innate immunity in overnutrition-induced neuroinflammation was observed. The activation process firstly targeted IKKβ activated by phosphorylation at S177 and S181, which triggered the phosphorylation, ubiquitination, and proteasomal degradation of its substrate IκBα. NF-κB was then released in combination with IκBα and transported to the nucleus to transcript its relative genes (Hayden and Ghosh, 2008). Interestingly, Zhang et al. (2008) proved that up-activated NF-κB in the hypothalamus was elevated by 2-folds after exposure to HFD, and mRNA levels of IKKβ and IκBα in the hypothalamus were also enhanced. Additionally, studies proposed TNF-α as an activator and a gene product of IKKβ/NF-κB (Cai, 2013). Furthermore, inhibited weight gain, glucose intolerance, and IR were found in HFD-fed mice with a tamoxifen-inducible astrocyte-specific knockout of IKKβ (Douglass et al., 2017).

High nutritional diet and obesity can lead to cognitive dysfunction through the process of neuroinflammation, as formerly discussed; therefore, some consider it as one of the mechanisms of diet and obesity-induced cognitive impairment.

### Neuroinflammation Affecting Cognitive Deficits

Microglia and astrocytes are the brain’s resident immune cells. As discussed above, overnutrition induces the expression of inflammatory cytokines as well as triggers microglia to a proliferated and activated state. Microglia surveil and phagocytose synapses regulate neural circuit formation and function during this process. During interaction with synapses, specific presynaptic (SNAP25) and postsynaptic (PSD95) proteins have been identified (Miyamoto et al., 2013; Miller and Spencer, 2014). Apart from structural reconstruction, microglia influence synaptic transmission, and functional maturation. LPS, a classic ligand of TLR4, activates microglia to release ATP, which works as a primary messenger to recruit astrocytes by purinergic signaling. Astrocytes then release glutamate to promote synaptic transmission. Furthermore, when microglia are inhibited by an anti-inflammatory called agentminocyclin, neuron remains silent to LPS stimulation, which further suggests that active microglia are indispensable to ALPS-mediated neuronal spontaneous excitatory postsynaptic currents (EPSCs) (Tikka and Koistinaho, 2001; Pascual et al., 2012).

In addition, systemically-derived pro-inflammatory cytokines also affect neuronal function, especially in neurodegenerative disorders. A meta-analysis showed elevated CSF TGF-β, MCP-1, and YKL-40 levels in AD patients and increased CSF TGF-β1, IL-6, and IL-1β levels in PD patients (Chen et al., 2018). TNFα is a vital neurodegenerative factor that is associated with M1 microglial polarization. Treating 12-month-old APP/PS1 mice with an ICV injection of anti-TNFα mAb triggered reductions in both Aβ1–42 load and plaque deposition. Similarly, by removing NLRP3 in APPSWE/PS1ΔE9 mice, age-dependent elevation in IL-1β was ablated, which alleviated the Aβ burden and rescued spatial learning and memory impairments (Minter et al., 2016).

Various medications have been proved to improve neuroinflammation. Atypical antidepressant agomelatine (Ago) could reverse gliosis in specific brain regions, which also augmented its curative effects on depression (Atanasova et al., 2021). Since circulating adiponectin (APN) deficiency could trigger neuroinflammatory reactions, the APN receptor agonist adipoRon was applied to produce anti-inflammatory effects by decreasing microglial and astrocyte activation as well as restricting cerebral cytokine levels, which is also reckoned as a potential treatment for AD (Ng et al., 2021). Besides, traditional medications such as honokiol are also involved in neuroprotection and neuroinflammatory alleviation (Talarek et al., 2017). Therapies targeting neuroinflammation probably improve psychiatric symptoms to some extent due to the close relationship between these symptoms with neuroinflammation and psychiatric diseases (Najjar et al., 2013; Meyer et al., 2020). More importantly, they may avoid the side effects of obesity or overweight caused by many typical psychiatric drugs. A large number of studies confirmed that antipsychotic drugs, mood stabilizers, and antidepressants had negative effects on weight gain, even for short-term usage (Wirshing, 2004; Chao et al., 2019). Therefore, for psychiatric patients with weight problems, periodic monitoring of BMI and substitute medications are necessary.

In conclusion, obesity and overnutrition stimulate central inflammation *via* various mechanisms, which can be presented in gliosis and many inflammatory mediators. In addition, increased central immune cells and cytokines target synaptic modification and pathology of neurodegenerative diseases, which negatively impact cognitive function.

## Conclusion and Future Direction

To sum up, numerous preclinical and clinical studies have proved the cognition-related behavioral changes, imaging alterations, and multiple mechanisms involved in overnutrition- and obesity-induced cognitive impairment. Crucially, it has been confirmed that overnutrition independently influenced brain IR, gut microbiota composition, and neuroinflammation activation before the onset of obesity. For example, short-term exposure to HFD induced brain IR and the activation of lIKKβ/NF-κB signaling. Diet exerts a great impact on the abundance and diversity of the gut microbiome and, through gut-brain bidirectional communication, influences the brain. Pro-inflammatory factors and central immune cells are sensitively activated even after one 1-day HFD to phagocytose synapses and deteriorate neural functions.

Furthermore, several interventions have been explored to ameliorate cognitive deficits, involving dietary modulation, physical exercise, and other mechanism-targeted drugs. Apart from shifting to Mediterranean diets well-proved to benefit cognition, caloric restriction (CR) and intermittent fasting (IF) regimes are other alternatives. In a preclinical trial, mice subjected to 20% CR had increased dendric spines and improved cognitive performance in Barnes maze and novel object recognition tests (Wahl et al., 2018). Overweight individuals with CR for 3 months also displayed memory uplift accompanied by a decline in plasma insulin and C-reactive protein levels (Witte et al., 2009). IF exerts comparable effects with CR, and their differences require further exploration (Seimon et al., 2015). Physical exercise cannot be neglected as an effective intervention. Several meta-analyses pointed out its multi-domain beneficial effects on cognition in older people and patients with AD (Gavelin et al., 2021; López-Ortiz et al., 2021). The gut microbiome–targeted therapies are extremely popular and have achieved a breakthrough in recent years, which include fecal microbiota transplantation (FMT), antibiotics, prebiotics, probiotics, synbiotics, and other products that can modify the intestinal microbial ecosystem. The role of FMT, prebiotics and probiotics in neural plasticity, anti-inflammation, or brain function has been supported by a fair amount of evidence (Solas et al., 2017). Microbiota-accessible carbohydrates (MACs) and β-glucan can also combat gut-brain dysfunction and prevent neurodegenerative disorders (Shi et al., 2020a,b). Besides, anti-inflammatory drugs such as non-steroidal anti-inflammatory drugs prevent the onset of AD, and anti-obesity medications regulate eating behavior through crosstalk between central neural signaling and gut hormones (Imbimbo et al., 2010; Clemmensen et al., 2017). Physical therapies such as repetitive transcranial magnetic stimulation (rTMS) can also improve cognitive function and loss of weight at the same time, but the related mechanisms still need to be revealed to achieve better benefits for patients (Chou et al., 2020).

However, gaps still exist in the lectures. For the overnutrition models, the diet is restricted to high fat or high sugar with few trials to high sucrose, high FFA, or high protein. It also lacks standards to define which percent higher can be called excessive intake. Although the effects of an overnutrition diet on brain function and behavior have been identified, the correlation between them is still not clearly understood. Many questions remain unsolved, for example, which of these causes more severe cognition deficits or exerts reversible impacts. From a mechanistic perspective, several underlying mechanisms have unraveled the relationship between the brain and diet or obesity. However, further studies are needed to dig into the key processes and identify whether the two-act in parallel or one is superior to the other. Besides, more attention should be paid to the interplay between various avenues. For example, an HFD accompanied by intestinal microbiota, especially gut pathobionts, can trigger chronic inflammation; the latter contributes to metabolic alterations such as IR (Caesar et al., 2015; Dabke et al., 2019).

Since the effect of diet or obesity on cognition and its underlying mechanisms vary according to internal and external factors, a comparison between race, ethnicity, sex, and age should be considered (Daly et al., 2022). For example, central and peripheral inflammation-related synaptic remodeling contributed to obesity-induced hypothalamic dysfunction. However, female obese mice lacked microglia activation and macrophage infiltration (Lainez et al., 2018). Male and female mice also exhibited different diet-induced microbiome alterations in certain genera (Daly et al., 2022). Additionally, age served as a detrimental factor in obesity-related cognition deficit by exacerbating neuroinflammation and BBB disruption (Tucsek et al., 2014a,b). However, mounting evidence was found in animal experiments. More clinical trials are needed to find out specific conditions under which the influences of diets are reversible or the effects of treatments are augmented.

Furthermore, obesity indices are expected to gradually evolve from simply BMI to more comprehensive measurements such as WC, WHR, and visceral adiposity (Olsthoorn et al., 2021). These parameters are conducive to classifying obesity subgroups with their different association with cognitive results. At the clinical level, if anthropometric indices of obesity can be standardized to assess the risk of cognition impairment, it may be expedient for both patients’ self-evaluation and doctors’ initial judgment.

Finally, longitudinal studies on multiple pathways such as IR, gut microbiome, and inflammation are required in the future, which may indicate the optimal time point for interventions to prevent, reverse, cure, or improve the negative effects of over-nutrition diets or obesity on cognitive impairments.

## Author Contributions

QZ and KJ drafted the manuscript. JL and YZ revised the draft manuscript. BC, RL, and SC reviewed and summarized the relative works of literature. All authors read and approved the final manuscript.

## Author Disclaimer

All claims expressed in this article are solely those of the authors and do not necessarily represent those of their affiliated organizations, or those of the publisher, the editors, and the reviewers. Any product that may be evaluated in this article, or claim that may be made by its manufacturer, is not guaranteed or endorsed by the publisher.

## Conflict of Interest

The authors declare that the research was conducted in the absence of any commercial or financial relationships that could be construed as a potential conflict of interest.

## Publisher’s Note

All claims expressed in this article are solely those of the authors and do not necessarily represent those of their affiliated organizations, or those of the publisher, the editors and the reviewers. Any product that may be evaluated in this article, or claim that may be made by its manufacturer, is not guaranteed or endorsed by the publisher.

## Abbreviations

AD: Alzheimer’s disease
ADC: apparent diffusion coefficient
Ago: agomelatine
AgRP: agouti-related peptide
AIDS: acquired immune deficiency syndrome
ANS: autonomic nervous system
APN: adiponectin
ARC: arcuate nucleus
Aβ: amyloid-β
BBB: blood brain barrier
BDNF: brain-derived neurotrophic factor
BMI: body mass index
CNS: central nerve system
CR: caloric restriction
CSF: cerebrospinal fluid
DCE-MRI: dynamic contrast enhanced magnetic resonance imaging
DII: dietary inflammatory index
DIO: diet-induced obesity
DTI: diffusion tensor imaging
ENS: enteric nervous system
FFA: free fatty acid
fMRI: functional magnetic resonance imaging
FMT: fecal microbiota transplantation
GABA: gamma-aminobutyric acid
GBC: global brain connectivity
GLP1R: glucagon-like peptide-1 receptor
GMV: gray matter volume
HAND: HIV-associated neurocognitive disorders
HFD: high fat diet
HPA: hypothalamic–pituitary–adrenal
ICV: intracerebroventricular
IF: intermittent fasting
IMD: immune-metabolic depression
IR: insulin resistance
IRS: insulin receptor substrate
LPS: lipopolysaccharides
MACs: microbiota-accessible carbohydrates
MAPK: mitogen-activated protein kinase
MBH: mediobasal hypothalamus
MCH: melanin-concentrating hormone
MCI: mild cognitive impairment
MDD: major depressive disorder
MetS: metabolic syndrome
MRI: magnetic resonance imaging
MyD88: myeloid differentiation primary response gene 88
NGAL: neutrophil gelatinase-associated lipocalin
OTUs: operational taxonomic units
PD: Parkinson’s disease
PET: positron emission tomography
PI3: phosphoinositide 3-kinase
POMC: pro-opiomelanocortin
PVN: paraventricular nucleus
rTMS: repetitive transcranial magnetic stimulation
SFA: saturated fatty acid
SPECT: single-photon emission computed tomography
SV2A: synaptic vesicle glycoprotein 2A
T2DM: type 2 diabetes mellitus
T2WI: T2-weighted imaging
TBF: total body fat
UFA: unsaturated fatty acid
VAT: visceral adipose tissue
WC: waist circumference
WHR: waist-to-hip ratio.
